# Barrett's esophagus: The pathomorphological and molecular genetic keystones of neoplastic progression

**DOI:** 10.1002/cam4.4447

**Published:** 2021-12-06

**Authors:** Ksenia S. Maslyonkina, Alexandra K. Konyukova, Darya Y. Alexeeva, Mikhail Y. Sinelnikov, Liudmila M. Mikhaleva

**Affiliations:** ^1^ Research Institute of Human Morphology Moscow Russian Federation

**Keywords:** Barrett's esophagus, epigenetic changes, esophageal cancer, molecular pathways, oncotransformation, preventative targets

## Abstract

Barrett's esophagus is a widespread chronically progressing disease of heterogeneous nature. A life threatening complication of this condition is neoplastic transformation, which is often overlooked due to lack of standardized approaches in diagnosis, preventative measures and treatment. In this essay, we aim to stratify existing data to show specific associations between neoplastic transformation and the underlying processes which predate cancerous transition. We discuss pathomorphological, genetic, epigenetic, molecular and immunohistochemical methods related to neoplasia detection on the basis of Barrett's esophagus. Our review sheds light on pathways of such neoplastic progression in the distal esophagus, providing valuable insight into progression assessment, preventative targets and treatment modalities. Our results suggest that molecular, genetic and epigenetic alterations in the esophagus arise earlier than cancerous transformation, meaning the discussed targets can help form preventative strategies in at‐risk patient groups.

## Graphical abstract

Several important keystone genetic events occur during carcinogenesis in the distal esophagus. These processes should be diagnosed during routine evaluation of patients with Barrett's esophagus to prevent disease progression and malignization. The aforementioned genetic aberrations are detected as early as 2 years before diagnosis of esophageal carcinoma.

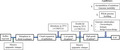

## 1 INTRODUCTION

Esophageal adenocarcinoma (EAC) is predominantly found in the distal third of the esophagus. Early diagnostics of EAC is challenging due to the lack of specific symptoms. More than 50% of EAC cases are diagnosed at stages III‐IV, which explains the poor prognosis associated with this malignancy. The recently reported 5‐year survival of patients with EAC is around 20.1%–23.4%.^1^, ^2^ Risk factors of EAC development include male gender, gastro‐esophageal reflux disease (GERD), Barrett's esophagus and smoking.^1^, ^3^, ^4^, ^5^, ^6^, ^7^, ^8^ Barrett's esophagus (BE) is a premalignant condition for EAC. Risk of developing EAC is significantly higher in patients with BE compared to the general population.^6^ Routine endoscopic surveillance with histopathological assessment in BE patients aims for early detection of neoplasia.^9^, ^10^, ^11^, ^12^ Detection of dysplastic BE and T1a stage of EAC prompts endoscopic treatment which delivers high 5‐year survival rates.^13^, ^14^ Nonetheless, the role of BE and different types of metaplasia in the distal esophagus region in progression to EAC is under discussion^15^ and existing algorithms of endoscopic surveillance are suboptimal because most of patients diagnosed with EAC do not have any history of BE.^16^, ^17^, ^18^ Analysis of existing information on the different types of esophageal metaplasia pathways and their contribution to development of EAC will help delineate possible diagnostic and therapeutic targets. Our essay is focused on morphological diagnosis, immunohistochemical (IHC) examination and molecular‐genetic methods for dysplasia detection and prediction of neoplastic progression.

All images presented in this study were obtained following approval by the ethics committee at the 31st State City Hospital of Moscow (№03‐19 from 06.12.2019). All patients included in the pathomorphological study provided informed written consent.

## 2 RISK OF EAC IN METAPLASTIC PROCESSES OF THE ESOPHAGUS

Long lasting reflux exposure in distal esophagus results in initiation of columnar‐lined esophagus. Cardiac type metaplasia is the earliest morphologic finding, although multitude of gland structure phenotypic variants arises in segment of metaplasia in distal esophagus over time.^19^, ^20^, ^21^ Proportion of glands goes through enteralization which causes development of intestinal metaplasia (IM, or so called specialized metaplasia) with easily found hallmark goblet cells (GCs) that are inserted among foveolar cells. Enteralization is believed to start with expression of immunohistochemical markers of intestinal differentiation in columnar epithelium, such as CDX2, villin and Das‐1,^22^, ^23^ followed by MUC2 expression and development of GCs. Paneth cells are detected in some cases of specialized metaplasia. Segment of metaplasia may also contain different variants of gastric metaplasia: glands of cardiac, oxynto‐cardiac and fundic type. Various phenotypes of metaplasia can be identified in biopsy pieces of distal esophagus separately or in combination.

There are two ultimately different approaches to BE diagnostics.^15^, ^24^ British Society of Gastroenterology (BSJ)^9^ and international consensus BOB CAT^10^ define BE as any type of columnar metaplasia in distal esophagus. Meanwhile, American Gastroenterological Association (AGA)^11^ and Russian Society of Pathologists (RSP)^12^ require mandatory presence of IM for diagnosis of BE because IM is associated with increased risk of EAC development.

**FIGURE 1:**
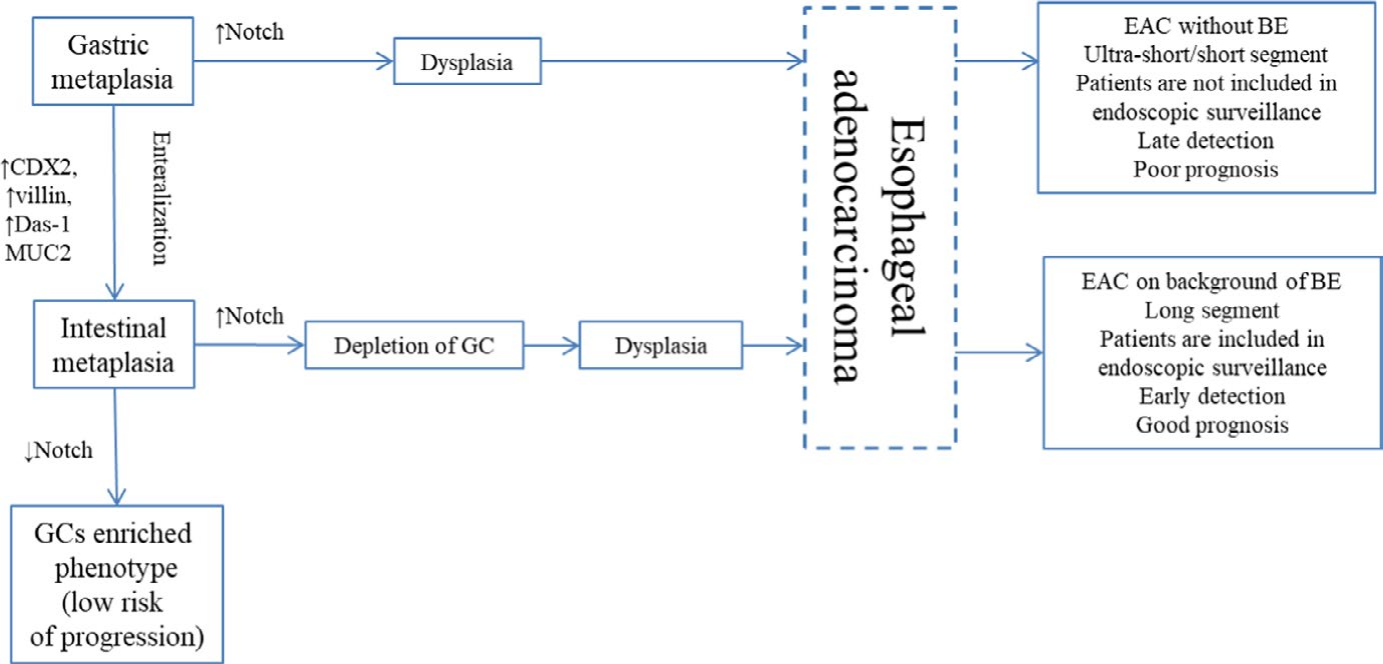
Schematic illustration shows suggested pathways of progression to EAC in gastric and intestinal metaplasia

For a long time, it was accepted that more than 90% of all EAC arise at background of IM.^11^, ^12^, ^25^ In a large epidemiological study, Bhat S. et al.^26^ identified incidence of high‐grade dysplasia (HGD)/EAC in patients with IM to be 0.38% a year, and only 0.07% a year in patients without IM (hazard ratio 3.54, 95% CI 2.09–6.00, *p* < 0.001), whereas in other research incidence of HGD/EAC did not differ in patients with IM and gastric metaplasia at initial biopsy.^27^, ^28^ Tan M.C. et al.^13^ demonstrated in meta‐analysis that BE (IM) is detected only in 56.6% patients (95% CI 48.5%–64.6%) at the time of EAC diagnosis. In addition, BE is more frequently identified in patients with early EAC: in studies, where early EAC was diagnosed in 100% of cases, BE was confirmed in 91.3% patients (95% CI 82.4%–97.6%). Sawas T. et al.^29^, ^30^ observed IM only in 45.0%–49.9% patients with EAC and the frequency of BE detection in patients with different stages of EAC was nearly equal that contradicts overgrowth of IM by tumor. Sawas T. et al.^29^, ^30^ identified two phenotypes of EAC with different prognosis based on the presence or absence of BE: EAC with BE at background was characterized by better prognosis than EAC without BE. The authors suppose ultra‐short segment of IM to be the source of EAC without BE. Nevertheless, it is widely accepted that the chance of IM detection rises with increase in segment length.^27^, ^31^, ^32^, ^33^, ^34^ Considering that IM is rare in ultra‐short segment (it is detected only in 14.8% patients^33^) and in most cases it comprises cardiac and oxynto‐cardiac metaplasia, it seems logical to assume the source of such EAC to be ultra‐short and short segments of gastric type metaplasia (Figure 1).

This proposition is supported by results of several studies. Takubo K. et al.^35^ demonstrated that more than 70% cases of minute EAC arise at background of cardiac or fundic‐type metaplasia surrounding the tumor. Performing IHC examination Watanabe G. et al.^36^ detected gastric phenotype (expression of gastric differentiation markers MUC5A and MUC6 with negative expression of intestinal markers) more frequently in minute tumors. Several phenotypes of dysplasia and EAC were identified based on IHC examination with gastric and intestinal markers that confirm presence of two distinct pathways in carcinogenesis: intestinal and foveolar,^37^, ^38^ although genetic analysis showed that both metaplasia types harbor the same mutations.^39^ Using histological, IHC examination and genetic analysis, Lavery D.L. et al.^40^ revealed that even when IM is present EAC arises from gastric type metaplasia. On the other hand, high density of GCs in BE is associated with a decrease in the risk of EAC development and may represent a protective mechanism of adaptation.^41^, ^42^, ^43^ Inhibition of Notch‐signaling causes proliferative cells in metaplastic glands to become terminally differentiated GC.^44^, ^45^ Thus, induction of GC differentiation may represent a potential therapeutic strategy of EAC prevention in patients with BE.^41^, ^44^

## 3 ENDOSCOPIC EVALUATION IN BE AND EAC: STANDARD PROCEDURE AND COMPUTER‐AIDED DETECTION (CAD)

White light endoscopy (WLE) with four‐quadrant biopsy each 2 cm plus biopsy from any suspicious visual lesions is recommended by most of the guidelines^9^, ^10^, ^12^ as an effective tool for dysplasia detection.^46^ Nevertheless, adherence to standard protocol is low, comprising between 24.1% and 82.7%^47^, ^48^, ^49^ and is even lower in long segment of dysplasia, where dysplasia is more likely to be found. Standard protocol is time and cost consuming, prone to sampling error and results in high load of pathologists with abundant biopsies.^16^ That is why a lot of different endoscopy modalities and techniques were tried for visualization of dysplasia and precise biopsy, among them narrow‐band imaging (NBI),^50^, ^51^, ^52^ acetic acid chromoendoscopy (AAC),^53^, ^54^, ^55^, ^56^ autofluorescence imaging (AFI),^57^, ^58^ confocal laser endomicroscopy (CLE)^59^, ^60^, ^61^, ^62^, ^63^ and volumetric laser endomicroscopy (VLE).^64^, ^65^, ^66^ Although some studies demonstrated different imaging modalities to be efficient, in other studies the use of these techniques did not report benefits in dysplasia detection rate.^67^, ^68^, ^69^, ^70^ Sensitivity of standard protocol with 4‐quadrant biopsy ranged from 28% to 85% in different studies and specificity varied from 56% to 100%, this led American Society for Gastrointestinal Endoscopy to set thresholds for any Preservation and Incorporation of Valuable endoscopic Innovations (PIVI)^71^: an imaging technology with targeted biopsies should have a per‐patient sensitivity of 90% or greater, negative predictive value (NPV) of 98% or greater for detecting HGD or early EAC and specificity of at least 80% to allow a reduction in the number of biopsies compared to standard protocol.

However, recent research showed benefits of CAD using WLE images^72^, ^73^, ^74^, ^75^, ^76^ in dysplasia and early EAC detection. At first, F. van der Sommen et al.^72^ used 100 WLE images obtained from 44 patients with BE to develop CAD model based on machine learning algorithm that identified HGD and early EAC with sensitivity of 86% and specificity of 87% at the patient level. Next, Mendel R. et al.^73^ performed a convolutional neural networks (CNN) analysis of BE using 50 WLE images of EAC and 50 BE images from an open access database (Endoscopic Vision Challenge MICCAI 2015) and achieved sensitivity of 94% and specificity of 88%. Notably, A.J. de Groof et al.^74^ developed a hybrid ResNet‐UNet model CAD system using 5 independent WLE endoscopy datasets. Pre‐training was performed using large series of 494,364 labelled endoscopic images. Then 1247 images of early neoplasia and non‐dysplastic BE (NDBE) were used in the second‐step training and other 297 images (3rd step) – for internal validation. Two sets (4th and 5th step) each of which containing 40 neoplastic and 40 NDBE images served for external validation. At the 5th step, accuracy was 88%, sensitivity 93% and specificity 83% that outperformed results of general endoscopists (73%, 72% and 74%, respectively). The computational speed for classification and delineation of the endoscopic images in this study was compatible for use in real time during endoscopic surveillance. Hashimoto R. et al.^75^ also developed CNN algorithm for detection of dysplastic BE and NDBE with sensitivity of 96.4%, specificity of 94.2% and accuracy of 95.4%. This study also suggested possibility of real‐time implementation. It was practically proved by Ebigbo A. et al.^76^ In this study, 129 endoscopic images were used for CAD system training and validation was performed in real‐time assessing images from 14 patients with further histological confirmation. In this study, CAD sensitivity of 83.7%, specificity of 100.0% and overall accuracy of 89.9% were reached. Few studies also assessed CAD dysplasia detection using VLE.^77^, ^78^, ^79^ The data are summarized in Table 1.

**TABLE 1:** Standard WLE and CAD models in diagnostics of dysplasia and EAC

Method	Advantages	Disadvantages	Articles	Number of patients/images	Sensi‐tivity	Speci‐ficity
WLE with standard 4‐quadrant biopsy	Is recommended by most of guidelines as effective	Poor adherence to protocol Prone to sampling error High load of pathology department	ASGE PIVI (2012)^71^	—	28%–85%	54%–100%
WLE + CAD	Helps to avoid subjectivity in evaluation. Less biopsy fragments taken Sensitivity and specificity is higher than general endoscopists reached. Time of processing is compatible with real‐time use.	Not currently used in general practice. Evaluation in real‐time needs to be developed and validated.	van der Sommen F. et al. (2016)^72^	44 patients (100 images)	86%	87%
			Mendel R. et al. (2017)^73^	100 images from MICCAI database	94%	88%
			de Groof A.J. (2020)^74^	Pre‐training ‐ 494,364 images Training – 1247 images Internal validation – 297 images External validation – 2 sets of images (80 + 80).	93%	83%
			Hashimoto R. et al. (2020)^75^	Training – 65 patients (1835 images) Validation – 458 images	96.4%	94.2%
			Ebigbo A. et al. (2020)^76^	Training ‐ 129 images Validation ‐ 14 patients (62 images)	83.7%	100.0%

## 4 WIDE AREA TRANSEPITHELIAL SAMPLING WITH COMPUTER‐ASSISTED THREE‐DIMENSIONAL ANALYSIS (WATS)

WATS represents esophageal brush biopsy that samples large circumferential area to obtain full‐thickness transepithelial tissue sample. Then computer‐assisted analysis using neural networks integrates up to 50 3‐μm optical slides to create a single three‐dimensional image of glands for pathology review.^80^ Several studies demonstrated that WATS significantly improved the detection of both BE and esophageal dysplasia (Table 2).^80^, ^81^, ^82^, ^83^, ^84^, ^85^ Thus, in a prospective multicenter community‐based study enrolling 12,899 patients, Smith MS et al.^80^ showed that adding WATS to routine forceps biopsy raised the yield of dysplasia detection from 0.68% to 2.33% and increased the overall detection of dysplasia by 242% (95% CI 191%–315%). Rate of BE detection by forceps biopsy was 13.1% and WATS raised it to 33% increasing the overall detection of BE by 153% (95% CI 144%–162%). In meta‐analysis, WATS as an adjunct to forceps biopsy yielded relative increase of 1.62 in detection of BE (95% CI 1.28–2.05, *p *< 0.0001) and relative increase of 2.05 in the detection rate of esophageal dysplasia (95% CI 1.42–2.98, *p *= 0.0001).^84^ WATS adjunct to the standard random 4‐quadrant forceps biopsies showed to be cost‐effective for screening of at risk patients.^86^

**TABLE 2:** Comparison of WATS technology with standard 4‐quadrant biopsy histological assessment

Diagnostic method	Advantages	Disadvantages	Sensitivity	Specificity	Rate of BE detection	Rate of dysplasia detection	κ‐value
4‐quadrant biopsy	Standard procedure Is recommended by most of guidelines as effective	Prone to sampling error. Time and labor intensive. High load of pathology department. Need for confirmation of dysplasia by second pathologist or expert in GI pathology. Only 3.5–5% of mucosa is evaluated.^89^	28–85%^71^	54–100%^71^	13.1%^80^	0.68%^80^	0.24–0.66^90^, ^91^, ^92^, ^93^
WATS	Improves dysplasia detection compared with 4‐qudrant biopsy alone. No complications reported. Commercially available Cost‐effective Good inter‐observer agreement	Not a separate method, but adjunct to routine 4‐quadrant biopsy. Not a routinely used method. Assessed in a single laboratory CDx Diagnostics (Suffern, NY). May be a source of dysplasia overdiagnosis.	96.9%^81^	52.3%^81^	33.0%^80^	2.33%^80^	0.86^87^

The inter‐observer agreement among pathologists in the diagnosis of dysplasia using WATS was better than for histopathology (Table 2). The overall mean kappa value for the 4 observers was calculated as 0.86 (95% CI 0.75–0.97). The kappa values for HGD/EAC, IND/LGD, and NDBE comprised 0.95 (95% CI 0.88–0.99), 0.74 (95% CI 0.61–0.85), and 0.88 (95% CI 0.81–0.94), respectively.^87^ Nonetheless, in forceps biopsy cytological atypia is assessed along with architecture changes. Therefore, WATS cannot substitute forceps biopsy, because it does not provide necessary information about architecture changes (for example, it cannot assess surface maturation required for diagnostics of dysplasia or differ glands at the bases of the pits that may mimic dysplasia) and invasion, but there is a concern that WATS may lead to overestimation of dysplasia.^88^

## 5 PATHOMORPHOLOGICAL FEATURES OF DYSPLASIA IN BARRETT'S ESOPHAGUS

**FIGURE 2:**
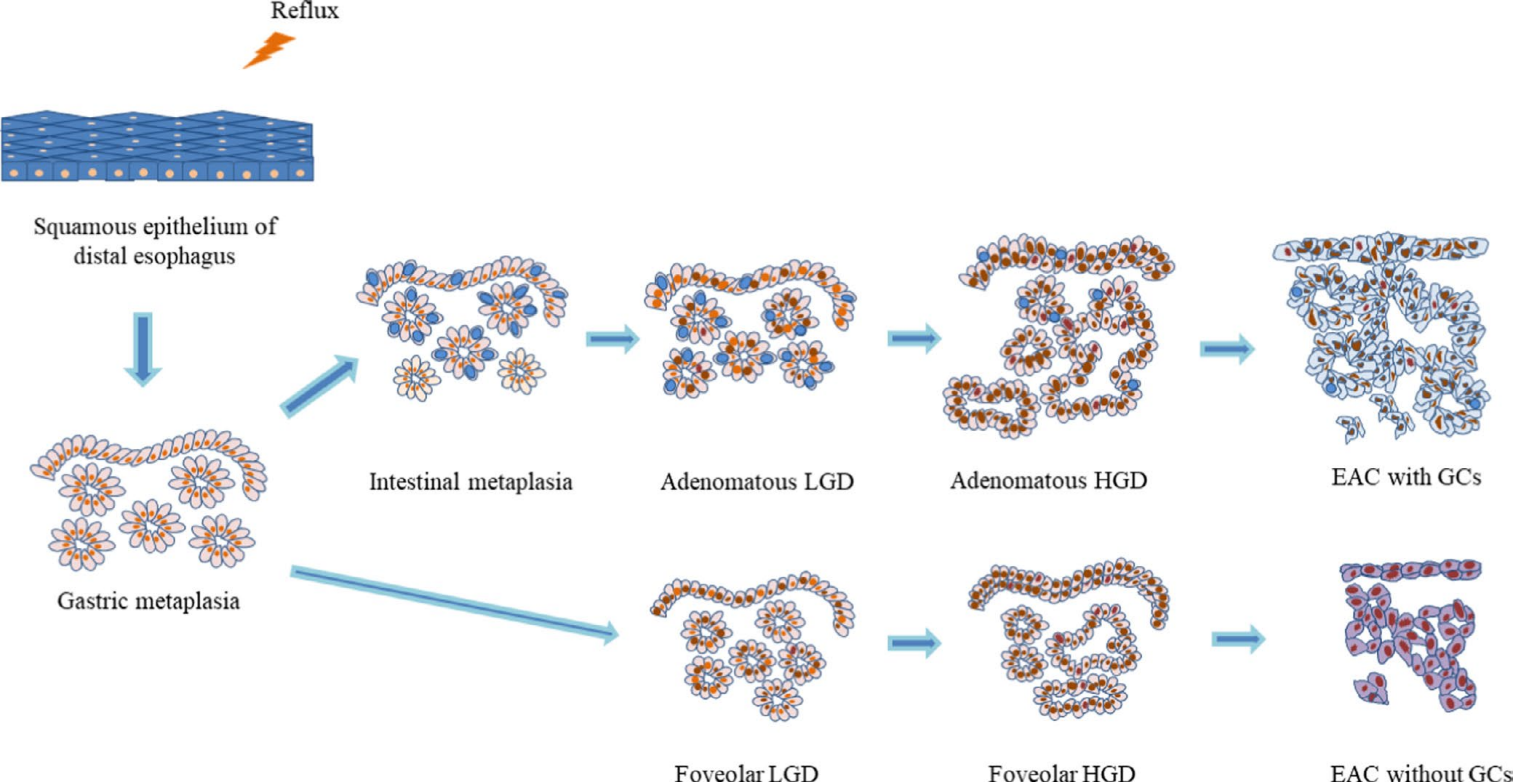
Schematic illustration that demonstrates changing morphological features during neoplastic progression in BE and non‐intestinal metaplasia of distal esophagus

Neoplastic progression in BE goes through the following stages: nondysplastic BE (NDBE)—low‐grade dysplasia (LGD)—HGD—EAC (Figure 2). Morphological detection of dysplasia in BE represents a clinically relevant factor for stratification of EAC development risk.^94^, ^95^, ^96^, ^97^, ^98^, ^99^, ^100^ The risk of EAC is 10‐fold higher in LGD compared with NDBE.^94^ Gradation of neoplastic changes at pathological examination is held in accordance with Vienna classification^101^ or criteria proposed by Reid B.J. et al.^102^ (Table 3). Both diagnostic systems are consistent with current clinical practice.^103^

**TABLE 3:** Comparison of two systems of dysplasia gradation in BE: proposed by Reid (1988) and the Vienna classification of gastrointestinal epithelial neoplasia (2000)

The Vienna classification of gastrointestinal epithelial neoplasia, 2000^101^	Consensus for grading dysplasia in BE, 1988^102^
Negative for dysplasia/neoplasia	Negative for dysplasia/neoplasia
Indefinite for dysplasia/neoplasia	Indefinite for dysplasia
Non‐invasive low‐grade neoplasia (low‐grade adenoma/dysplasia)	Low‐grade dysplasia
Non‐invasive high‐grade dysplasia High‐grade dysplasia Non‐invasive adenocarcinoma (carcinoma in situ) Suspicious for invasive carcinoma	High‐grade dysplasia
Invasive neoplasia Intramucosal adenocarcinoma Submucosal adenocarcinoma or beyond	Adenocarcinoma Intramucosal adenocarcinoma Invasive adenocarcinoma

Four morphological criteria were developed for dysplasia identification^90^, ^104^: (1) surface maturation versus epithelium in the glands, (2) architecture of glands, (3) cytological features of proliferation, and (4) presence of inflammation, ulcers or erosions.

**FIGURE 3:**
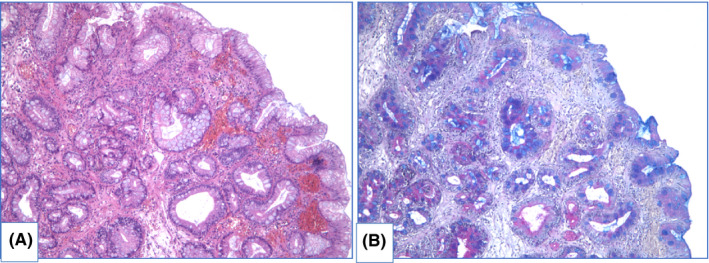
Nondysplastic BE. Specimen of IM in distal esophagus with high density of goblet cells, stroma shows inflammatory infiltration and extravasation: (A) hematoxylin and eosin staining, (B) PAS/Alcian blue staining, magnification ×100

**FIGURE 4:**
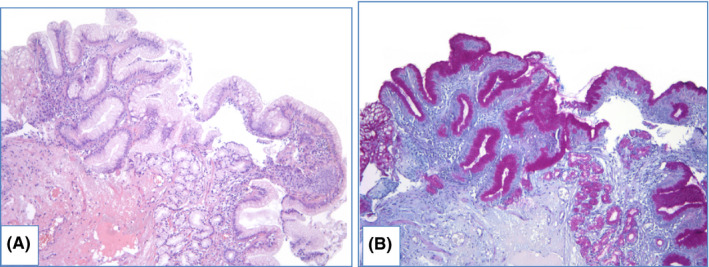
Gastric metaplasia with pseudo‐GCs in distal esophagus. Specimen of metaplastic distal esophagus with distended foveolar cells, containing apical mucus at the surface. (A) hematoxylin and eosin staining, (B) PAS/Alcian blue staining: cytoplasm of epithelial cells stains purple, magnification ×100

**FIGURE 5:**
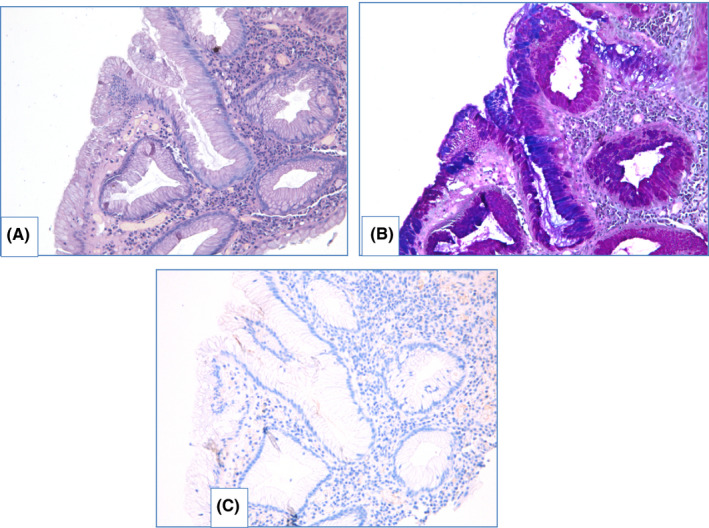
Pseudogoblet cells in gastric metaplasia. Specimen of columnar‐lined esophagus with elongated distended cells at the surface with apical mucus. (A) hematoxylin and eosin staining, (B) PAS/Alcian blue staining: cytoplasm of surface epithelium stains blue, (C) IHC evaluation with MUC2 shows negative expression, magnification ×200

NDBE specimens of esophageal mucosa are lined with columnar epithelium with round‐shaped glands containing GCs, surface maturation is obvious, extent of mixed inflammatory infiltration in stroma varies greatly (Figure 3). GCs are necessary to distinguish with pseudogoblet cells (pseudo‐GCs)—foveolar cells distended by mucus.^105^, ^106^ In most cases, it can be done in specimens stained with hematoxylin and eosin. GCs are more round in shape, with clear to bluish cytoplasm and triangle nuclei, and they are scattered through epithelium, whereas pseudo‐GCs are more elongated, with homogenous clear to pink cytoplasm and are organized in linear groups. In difficult cases, PAS/Alcian blue stain can be used to distinguish GCs and pseudo‐GCs. PAS/Alcian blue stains blue cytoplasm of GCs, whereas cytoplasm of pseudo‐GCs in most cases stains purple (Figure 4), although sometimes cytoplasm of pseudo‐GCs stains blue by PAS/Alcian blue like cytoplasm of GCs. In such cases, IHC examination with MUC2—a highly specific marker of GCs—is of value^107^ (Figure 5). At the other hand, Srivastava et al.^106^ stated that ancillary stains are not necessary in diagnosis of BE, because they do not add accuracy in GCs detection.

**FIGURE 6:**
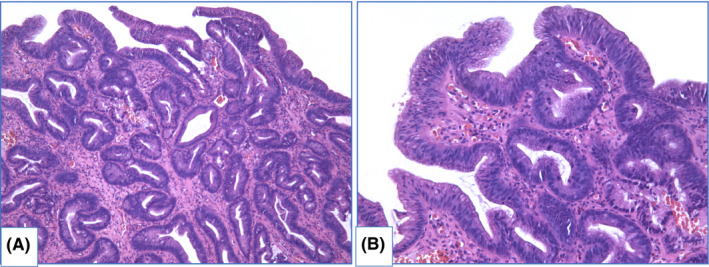
Adenomatous low‐grade dysplasia, hematoxylin and eosin staining: (A) magnification ×100, (B) magnification ×200. Specimen of columnar‐lined esophagus with lack of surface maturation. Most of glands are simple, round or angulated, few of them are dilated. Nuclear stratification and enlarged nucleo‐cytoplasmic ratio is obvious. Nuclei are pencillated, located in basal ½ of cells, mitoses are readily identified

**FIGURE 7:**
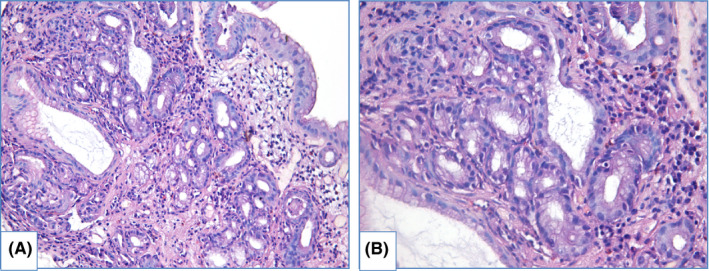
Foveolar low‐grade dysplasia, hematoxylin and eosin staining: (A) magnification ×200, (B) magnification ×400. Surface maturation is absent. Glands are mainly round shape, lined with cuboid epithelium with increased nucleo‐cytoplasmic ratio. Nuclei are round and hyperchromatic, with nucleoli. Few mitoses as well as apoptotic bodies are identified

There are two main types of dysplasia: more common adenomatous and rare foveolar.^108^, ^109^, ^110^, ^111^, ^112^ LGD shows weak or absent surface maturation. Inflammatory infiltration of stroma is scarce. Mild architecture distortion is typical: glands are slightly crowded, round and angulated, lined with columnar epithelium with nuclei located at the basal ½ of cells, and few nuclei may contain nucleoli. In adenomatous dysplasia (Figure 6), nuclei are mildly enlarged, slightly elongated, stratified and hyperchromatic, with few mitoses. In foveolar dysplasia, epithelial cells are cuboid with round to oval, and nuclei are slightly enlarged with hyperchromatosis (Figure 7).

**FIGURE 8:**
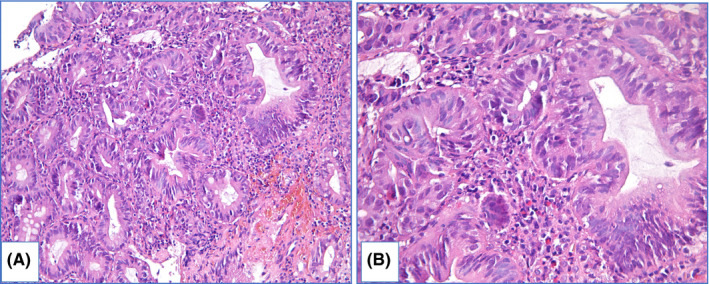
Adenomatous high‐grade dysplasia, hematoxylin and eosin staining, (A) magnification ×200, (B) magnification ×400. Specimen of columnar‐lined esophagus with complex structure of glands, including dilated glands with micropapillae. Nuclei of epithelial cells are prominently enlarged, elongated and hyperchromatic. Mark nuclear stratification and loss of polarity are also features of HGD

**FIGURE 9:**
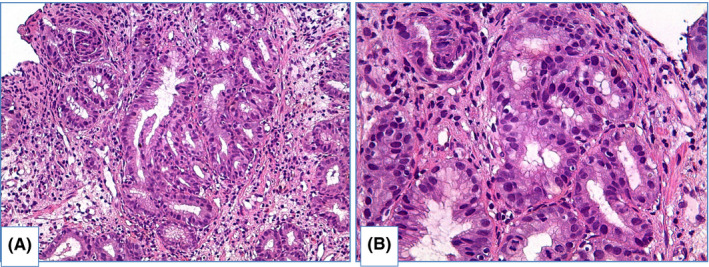
Foveolar high‐grade dysplasia, hematoxylin and eosin staining, (A) magnification ×200, (B) magnification ×400. Glands are predominantly round in shape, highly crowded, lined with columnar epithelium. Nuclei are round to oval, with severe enlargement, hyperchromatosis and a number of mitoses

HGD is characterized by prominent changes in architecture and/or pronounced features of cytological atypia as well as absent surface maturation. In adenomatous HGD (Figure 8) glands are crowded, with “back‐to‐back” appearance, and stroma between glands is scarce. Glands are of irregular shapes, some glands may be distended, and few glands may represent micropapillary or cribriform pattern. Loss of cellular polarity and prominent nuclear stratification is identified. Nucleo‐cytoplasmic ratio is highly increased, nuclei are elongated (pencil‐like), hyperchromatic, nuclear membrane is irregular, nucleoli may be easily found. Mitoses, including atypical ones, are readily identified. Foveolar HGD (Figure 9) harbors less extensive architecture changes but severe enlargement of nuclei, hyperchromatosis and noticeable nucleoli.

LGD and HGD are distinguished based on severity of (1) architecture distortion and (2) cytological atypia.^90^, ^104^, ^105^ In subset of cases, prominent cytological atypia with markedly enlarged, stratified, pleomorphic nuclei and a lot of mitoses is sufficient for diagnosis HGD even if changes in architecture are moderate. Prominent architecture distortion even accompanied with mild cytological atypia should be classified as HGD. In biopsy specimens with LGD count of GCs varies greatly—from few GCs to high density GCs. Although depletion of GCs is typical for dysplasia in general, Bansal et al.^31^ found association between LGD and high count of GCs. In HGD and EAC, count of GCs is usually decreased.

**FIGURE 10:**
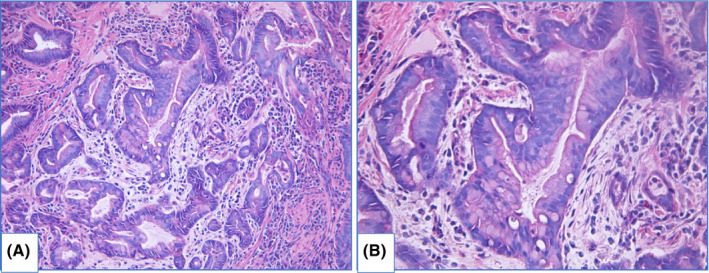
Invasive adenocarcinoma of distal esophagus: specimen of malignant tumor with glandular architecture, inflammatory infiltration and prominent desmoplasia, hematoxylin and eosin staining: (A) magnification ×200, (B) magnification ×400

Intramucosal EAC is diagnosed when there is invasion through the basal membrane into the lamina propria but not deeper than muscularis mucosae and invasive carcinoma is characterized by deeper invasion. In intramucosal EAC glands acquire “back‐to‐back” appearance, syncytial growth pattern and single cells or small clusters within the lamina propria. At this stage desmoplasia is either absent or subtle. Obvious desmoplasia and infiltrative growth pattern appear in tumors with deeper invasion (Figure 10).^113^, ^114^

Differential diagnosis of HGD and EAC in biopsy specimens is problematic with intraobserver agreement at about 0.30–0.65.^90^, ^115^, ^116^, ^117^ In early studies, when HGD was an indication to operative treatment, EAC was identified in 40%–70% esophagectomies after pre‐operative diagnosis HGD.^118^, ^119^, ^120^ Several features when they are identified in HGD are suspicious of unsampled EAC, including extensive cribriforming, dilated glands filled with necrotic debris, ulceration, intraluminal neutrophils and pagetoid pattern of neoplastic cells extension into squamous epithelium.^114^

**FIGURE 11:**
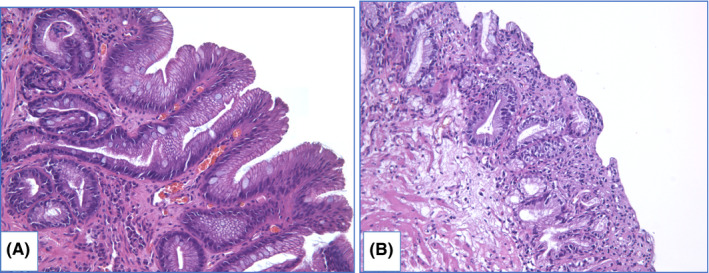
Indefinite for dysplasia, hematoxylin and eosin staining, magnification ×200: (A) fragment of columnar‐lined esophagus with artificial changes, angulated glands and slightly enlarged nuclei of epithelial cells, (B) fragment of columnar‐lined esophagus without surface epithelium with glands of irregular shapes, nuclei of epithelial cells are enlarged and focally hyperchromatic

In some observations it is troublesome to judge about dysplasia: morphological features are suspicious for dysplasia, but not sufficient to be definite.^105^, ^113^, ^121^ In these cases the appropriate diagnosis is indefinite for dysplasia—IND (Figure 11). Such situations derive from technical issues causing artificial changes, lack of surface epithelium or scarce biopsy pieces. Also IND may be diagnosed in specimens with abundant inflammation, ulcers or erosions resulting in reactive changes of epithelium that display focal weak surface maturation and cytological atypia (increased nucleo‐cytoplasmic ratio, hyperchromatosis and mitoses).

Incidence of EAC in patients with NDBE is estimated as 0.12%–0.33% a year.^26^, ^122^, ^123^ Rate of EAC detection increases with duration of surveillance and represents 0.19% a year in first 5 years after BE was diagnosed and 0.63% a year after 20 years of surveillance.^124^ Incidence of EAC in patients with LGD varies from 0.76 to 28% a year.^26^, ^98^, ^99^ The main reason for such a variety involves low intra‐observer agreement and poor reproducibility in diagnostics of presence and grade of dysplasia.^90^, ^91^, ^92^, ^99^, ^100^ At least two pathologists should independently perform histological examination in each case to avoid subjectivity in dysplasia detection.^9^, ^10^, ^11^, ^12^, ^120^, ^125^ In several studies, the number of pathologists that confirmed dysplasia was associated with rate of progression.^92^, ^93^, ^98^, ^120^, ^126^ Curvers W.L. et al.^126^ estimated incidence of HGD/EAC as 13.4% when initial diagnosis LGD was confirmed by expert pathologist and only as 0.49% in cases when expert pathologist downgraded the lesion to NDBE. In a prospective study of Duits L.C. et al.^98^ risk of progression to HGD/EAC increased 10‐fold when one pathologist established LGD, 27‐fold when two pathologists recognized dysplasia and 47‐fold when all three pathologists confirmed LGD. Nevertheless LGD is overdiagnosed in 28%–85% of observations,^98^, ^99^, ^126^ and HGD—in 40% of cases,^99^, ^127^ that leads to more aggressive treatment. IHC evaluation provides an opportunity not only to increase reproducibility of dysplasia diagnostics, but also to identify patients who are at high risk of neoplastic progression.

## 6 IMMUNOHISTOCHEMICAL MARKERS OF DYSPLASIA AND PROGRESSION PREDICTORS IN BE

**FIGURE 12:**
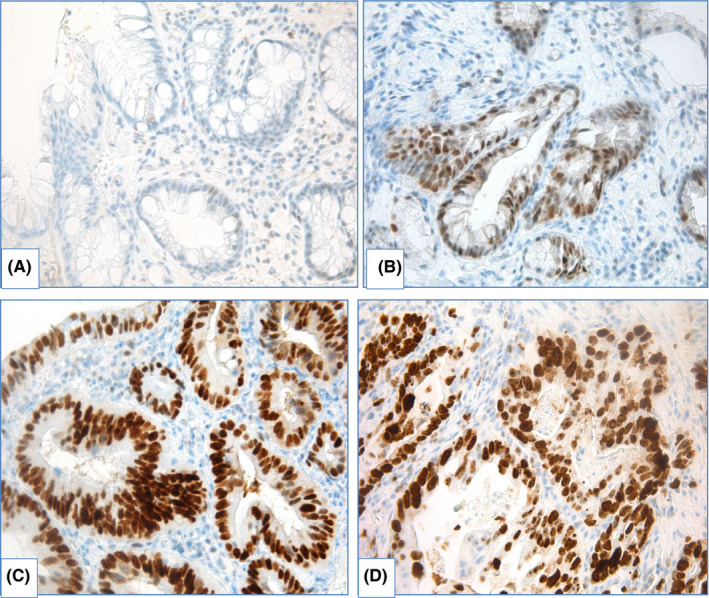
IHC examination with p53 in BE, magnification ×400. (A) nondysplastic BE: scattered expression of p53, (B) BE with LGD: moderate expression of p53 in proportion of epithelial cells, (C) BE with HGD: overexpression of p53, (D) EAC: overexpression of p53

*IHC with p53*. Inactivation of p53 is a key feature that occurs early in BE carcinogenesis,^128^, ^129^, ^130^ though it is not surprising that IHC evaluation with p53 is used for precise diagnostics of dysplasia. Two patterns of aberrant p53expression are identified: more frequently detected p53 overexpression (Figure 12) is associated with missense mutation of *TP53*, whereas absent p53 expression is caused by deletion or truncating mutation of *TP53*.^93^ Use of IHC evaluation with p53 improves reproducibility in morphological assessment of BE specimens and aids to avoid overdiagnosis of dysplasia.^92^, ^93^, ^131^, ^132^, ^133^

Moreover, aberrant expression of p53 is associated with increased risk of progression to EAC.^92^, ^133^, ^134^, ^135^, ^136^, ^137^, ^138^, ^139^, ^140^, ^141^, ^142^, ^143^, ^144^, ^145^ Murray L. et al.^135^ showed that diffuse expression of p53 is a predictor of progression to HGD/EAC (odds ratio – OR 8.42 [95% CI 2.37–30.0]), although p53 alone is not a reliable marker as in 2/3 of patients who progressed to HGD/EAC pattern of p53 expression was normal. In other studies OR of development HGD/EAC in aberrant p53 expression varied from 3.0 to 21.6.^139^, ^142^ Kastelein F. et al.^138^ demonstrated that prognostic value to predict neoplastic progression increased from 15% for morphological diagnosis of LGD to 33% for LGD with aberrant expression of p53.

In a prospective study, Younes M. et al.^141^ detected progression to HGD/EAC in 31.25% patients with expression of p53 in aggregates of epithelial cells and in 75% patients with p53 expression in multifocal aggregates of epithelial cells at initial biopsy (Kaplan–Meier analysis, *p* < 0.0001). In this study progression to HGD/EAC was seen in 40% observations with overexpression of p53 and only 0.3% patients with negative expression of p53 (Kaplan–Meier analysis, *p* < 0.0001).

Different definitions of aberrant IHC staining with p53 were used in various studies that make them difficult to compare. Although relevant association of aberrant p53 expression with neoplastic progression in BE was proved in meta‐analyses. Janmaat V.T. et al.^143^ estimated overall OR of progression to HGD/EAC in aberrant expression of p53 as 3.86 (95% CI 2.03–7.33), whereas in patients with NDBE with aberrant expression, overall OR comprised 6.12 (95% CI 2.99–12.52) and in patients with LGD it was as high as 8.64 (95% CI 3.62–20.62). More stringent criteria for aberrant staining definition resulted in higher overall OR.^143^ In other meta‐analysis performed by Snyder P. et al.^144^ OR of neoplastic progression in patients with aberrant expression of p53 in case–control studies varied from 3.84 to 5.95, as well as hazard ratio in cohort studies was estimated as 14.25 and 17.31 in different statistical models.

Use of IHC examination with p53 in routine practice was recommended by BSG^9^ and European Society of Gastrointestinal Endoscopy (ESGE).^134^

**FIGURE 13:**
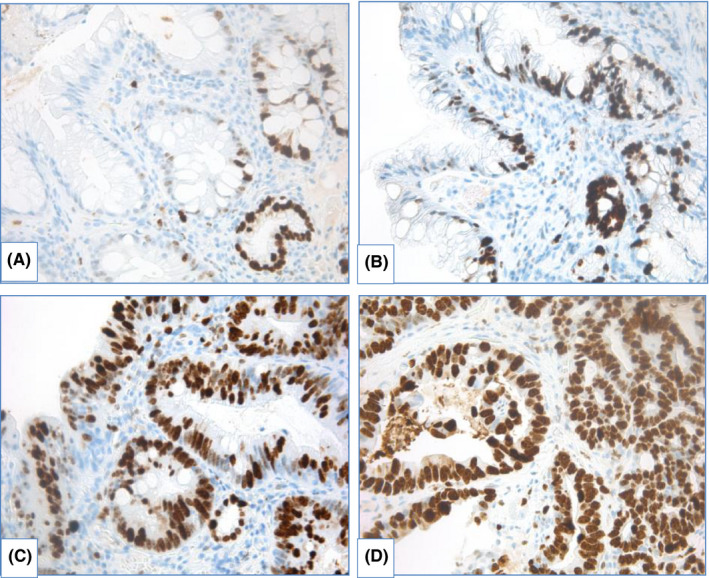
IHC evaluation with Ki67 in BE, magnification ×400: (A) nondysplastic BE: nuclear expression of Ki67 in the middle 1/3 of crypts, (B) BE with LGD: expression of Ki67 in the middle and the upper 1/3 of crypts, (C) BE with HGD: expression of Ki67 at the surface, (D) EAC: diffuse expression of Ki67

*IHC with Ki67*. Level of Ki67 expression that characterizes proliferative activity of cells increases in line: NDBE—LGD—HGD—EAC.^136^, ^137^, ^142^, ^146^, ^147^ Expansion of Ki67‐positive epithelial cells from proliferative zone at the middle third of crypts to surface is observed during neoplastic progression (Figure 13).^148^, ^149^, ^150^, ^151^, ^152^ Diffuse positive immunostaining of Ki67 at the surface is usually detected in HGD that helps us to distinguish HGD from LGD, where only minority of surface epithelium shows Ki67 expression.^136^, ^149^, ^150^ Use of IHC with Ki67 improves reproducibility of dysplasia diagnosis in BE.^131^, ^147^, ^153^ Extensive expression of Ki67 is also associated with progression to HGD/EAC.^136^, ^137^, ^145^

**FIGURE 14:**
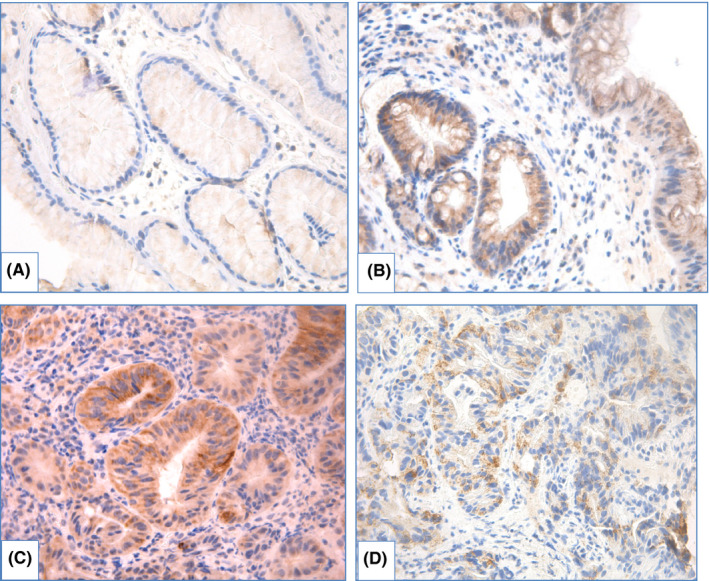
IHC with AMACR in BE, magnification ×400: (A) BE without dysplasia: weak attenuated expression in cytoplasm (background expression), (B) BE with LGD: granular expression of AMACR in proportion of epithelial cells, (C) BE with HGD: granular expression of AMACR in majority of epithelial cells, (D) EAC: granular expression of AMACR in proportion of epithelial cells

*IHC with AMACR*. The most controversial results were obtained for use of AMACR. In several studies, staining of AMACR was either absent^154^, ^155^, ^156^ or was detected in few cases of NDBE.^151^ Frequency of detection and extension of AMACR expression rises in line LGD—HGD—EAC (Figure 14).

Shi X.Y. et al.^151^ estimated sensitivity of AMACR expression for distinguishing between NDBE and dysplastic BE as 72.4% and specificity as 94.8%; staining of AMACR correlated with expression of p16, cyclin D1 and Ki67. Staining of AMACR was helpful to distinguish NDBE from IND/LGD and LGD from HGD. In other research, expression of AMACR did not differ between NDBE, IND and LGD, but was elevated in HGD.^157^ Sensitivity of AMACR expression varied widely: from 38 to 91.3% for LGD, from 64 to 95.8% for HGD and from 72 to 96% for EAC and specificity comprised 100%.^154^, ^155^, ^156^ Nevertheless, Strater J. et al.^158^ showed weak expression of AMACR in 83% cases of NDBE, indicating low sensitivity of AMACR in BE‐associated dysplasia detection.

In case–control study with large amount of samples (12,127 biopsies derived from 635 patients), Kastelein F. et al.^159^ demonstrated that strong AMACR expression was associated with progression to HGD/EAC (relative risk 4.8, 95% CI 1.9–12.6), although positive predictive value of strong AMACR expression (22%) was too low to use AMACR as the only marker of progression.

To sum up, IHC examination with p53, Ki67 and AMACR aims for precise diagnostics of dysplasia in BE (Table 4). Moreover, expression of these IHC markers has some prognostic value (Table 5), although predictive value of any IHC marker alone is limited. New challenge is to develop a combination of IHC markers for precise diagnostics of dysplasia in BE and prediction of progression.

**TABLE 4:** Histological evaluation and immunohistochemical assay in diagnostics of BE

Diagnostic method	Markers	Advantages	Disadvantages
Histopathology assessment of forceps biopsy	Presence and grade of dysplasia	Standard diagnostic procedure Routinely used Cost‐effective Easy to perform LGD histology is associated with progression	Poor inter‐observer agreement Low reproducibility High rate of dysplasia overdiagnosis Need for second opinion/evaluation by expert
IHC evaluation	p53	Confirming presence or absence of dysplasia Proved efficient in diagnostics Low cost Prognostic tool Recommended as a routine method by BSG^9^ and ESGE^125^ Extensively studied marker	Lack of standardization in interpretation of staining: different definitions and cut‐points are used in various studies.^106^ Although some studies demonstrate good inter‐observer agreement.^93^, ^138^ Positive staining is observed in up to 10% of NDBE^105^, ^106^ Although aberrant expression is highly associated with progression, proportion of patients with scattered staining also develops EAC^135^
	Ki67	Additional tool to evaluate proliferative activity Some data suggest association with progression Is available in routine practice Low cost	Nonspecific marker that stains both dysplasia and reactive epithelium Low value as a predictive marker
	AMACR	Additional tool to assess dysplasia in BE Has some prognostic value Is available in routine practice Low cost	Sensitivity and specificity varies greatly in different studies Low value as a predictive marker

**TABLE 5:** Histological evaluation and immunohistochemical assay predicting progression in BE

Markers	Article	Number of patients/Progressors^a^ (samples)	HR	RR	OR	Sens.	Sp.	PPV	NPV
LGD	Sikkema M. et al. (2009)^137^	54 patients/27 progressors (434 samples)	3.6; 95% CI 1.6–8.1						
	Kaye P.V. et al. (2009)^92^	175 patients/51 progressors				78%	80%	42%	95%
						(For consensus LGD)			
	Sikkema M. et al. (2011)^95^	713 BE patients/26 progressors		9.7; 95% CI 4.4–21.5					
	Kastelein F. et al. (2013)^138^	635 BE patients/49 progressors		4.2; 95% CI 2.4–7.3		44%	78%	15%	
	Moyes L.H. et al. (2016)^94^	722 BE patients/58 prevalent LGD	10.8; 95% CI 5.9–18.1 for progression to HGD; 7.3; 95% CI 3.6–14.7 for progression to EAC						
	Duits L.C. (2017)^98^	255 LGD patients/45 progressors			9.28; 95% CI 4.39–19.64 for persistent LGD				
	Duits L.C. et al. (2019)^142^	260 patients/130 progressors			7.5; 95% CI 1.7–32.8				
	Song K.Y. et al. (2020)^96^	69 LGD patients/16 progressors			4.18; 95% CI 1.03–17.1 for persistent LGD				
p53	Murray L. et al. (2006)^135^	210 patients/29 EAC and 6 HGD			11.7; 95% CI 1.93–71.4				
	Sikkema M. et al. (2009)^137^	54 patients/27 progressors (434 samples)	6.5; 95%CI 2.5–17.1						
	Kaye P.V. et al. (2009)^92^	175 patients/51 progressors				80%	68%	70%	78%
	Kasterlein F. et al. (2013)^138^	635 BE patients/49 progressors		6.2; 95%CI 3.6–10.9		49%	86%		
	Davelaar A.L. et al. (2015)^139^	116 patients/91 patients at follow‐up/11 progressors	17; 95% CI 3.2–96			63.6%	92.5%	53.8%	94.9%
	Horvath B. et al. (2016)^140^	103 patients/79 patients at follow‐up without prevalent neoplasia/4 progressors	12; 95% CI 1.43–100						
	Duits L.C. et al. (2019)^142^	260 patients/130 progressors			2.8; 95% CI 1.5–5.1				
	Altaf K. et al. (2017)^145^	Meta‐analysis (7415 samples)			10.23; 95% CI 7.19–14.55	60%	82%		
	Janmaat V.T. et al. (2017)^143^	Meta‐analysis (1322 patients/278 progressors)			3.18; 95% CI 1.68–6.03				
	Snyder P. et al. (2019)^144^	Case‐control studies: 1435 patients/209 progressors Cohort studies: 582 patients/28 progressors	Fixed‐effect model: 17.31; 95% CI 9.35–32.08 Random‐effect model: 14.25; 95% CI 6.76–30.02		Fixed‐effect model: 3.84; 95% CI 2.79–5.27 Random‐effect model: 5.95; 95% CI 2.68–13.22				
LGD +p53	Skacel M. et al. (2000)^133^	16 LGD patients/8 progressors				88%	75%		
	Kastelein F. et al. (2013)^138^	635 BE patients/49 progressors		11.2; 95%CI 5.7–22.0				33%	
Ki67	Sikkema M. et al. (2009)^137^	54 patients/27 progressors (434 samples)	5.2; 95% CI 1.5–17.6						
	Altaf K. et al. (2017)^145^	Meta‐analysis (1243 samples)			5,54; 95% CI 3.40–9.05	82%	48%		
AMACR	Kasterlein F. et al. (2013)^159^	635 patients/49 progressors (12,127 samples)		4.8; 95% CI 1.9–12.6		10%	96%	22%	91%

^a^ Progressors were defined as cases of HGD and EAC.

## 7 MACHINE LEARNING ALGORITHMS IN DIGITAL PATHOLOGY

To overcome low inter‐observer agreement on dysplasia diagnosis, attempts were made to develop machine learning approach applying to high‐resolution digital images with evaluation of morphometric and immunoquantitative parameters to distinguish between NDBE, dysplastic BE and EAC (Table 6).^146^, ^160^, ^161^, ^162^, ^163^, ^164^ The earliest work in this field was the study of Polkowsky W. et al. (1998)^160^ which suggested that quantitative assessment of cytometric and morphometric features associated with proliferation and differentiation could help in interpretation of BE histology. Combination of stratification index (SI) and Ki67 quantitative analysis gave the best classification result, but quantitation of p53 area added no value. Van Sandick J.W. et al.^161^ also showed benefit of SI and Ki67 area combination for distinguishing between LGD and HGD (91% correct classification), although combination of SI and p53 area was superior for distinguishing between NDBE and LGD (89% correct classification). Importantly, Baak J.P. et al.^146^ reported only 35% agreement between pathologists and experts. Experts downgraded high proportion of lesions due to severe inflammation, reactive changes, ulcers, proximity to squamo‐columnar junction and tangential cutting. In adequate sections morphometrical classification was closer to experts' grading (75% of agreement compared with 53% for pathologists). Sabo E. et al.^162^ developed neural network algorithm (NNET) for dysplasia grading using nuclear appearance (size, shape, chromatin texture, pleomorphism, symmetry and pseudostratification) that was able to correctly classify 89% of cases in distinguishing between NDBE and LGD and 87.5% of cases in differentiation between LGD and HGD. Moreover, in this study some of the variables were predictive for progression. Recently, Tomita N. et al.^163^ proposed new attention‐based network model that classified NDBE, dysplastic BE and EAC with mean accuracy of 0.83.

**TABLE 6:** Machine learning in diagnostics of BE

Article	Number of patients	Tissue material	Staining	Number of images/areas	Agreement between pathologists	Equipment	Classes	Parameters	Results
Polkowsky W. et al. (1998)^160^	35	Resection specimens after esopha‐gectomies	HE^a^ Ki67 p53	73 areas (58 – training set, 9 – second set, 6 – couldn't be assessed)	79%	QPRODIT1 version 6.1 (Leica Imaging Systems Ltd., Cambridge, UK)	NDBE LGD HGD ImCA^b^	Mean nuclear area (MNA) Mean nuclear volume (MNV) Mitotic activity index (MAI) MAI in the upper half of mucosa (MAI Up) Stratification index (SI) Ki67 area Ki67 area Up p53 area	Combination of SI and Ki67 area was the most valuable to discriminate between NDBE and LGD and between LGD and HGD (both – 94% of correctly classified areas). Discrimination between HGD and ImCA was lower than 80% of correct classification with any parameters
van Sandick J.W. et al. (2000)^161^	18	Biopsy specimens	HE Ki67 p53	105 areas derived from 371 biopsies	63%	QPRODIT1 version 6.1 (Leica Imaging Systems Ltd., Cambridge, UK)	NDBE LGD HGD	MNA MNV MAI SI Ki67 area p53 area	Combination of SI and p53 area helped to distinguish between NDBE and LGD (89% of correctly classified areas). Combination of SI and Ki67 area allowed discriminating between LGD and HGD (91% of correctly classified areas). Combination of SI, Ki67 area and MNV gave advantage in discriminating LGD and HGD (94% of correctly classified areas).
Baak J.P. et al. (2002)^146^	—	Biopsy specimens	HE Ki67	143 specimens	35% with experts	—	NDBE IND LGD HGD	SI MNA Ki67 area	Agreement between morphometric model and experts reached 75%.
Sabo E. et al. (2006)^162^	152 (97 for training, 55 for validation)	Biopsy specimens	HE	Not mentioned	Not mentioned	Image Pro Plus version 5.1 software (MediaCybernetics, MD, USA)	NDBE IND LGD HGD	Nuclear size Nuclear shape Nuclear chromatin texture Nuclear pleomorphism Nuclear symmetry Nuclear pseudostratification	The neural network algorithm (NNET) correctly classified 86% of the cases in distinguishing between NDBE and LGD (70% of NDBE and 95% of LGD) and 87% of cases in distinguishing between the LGD and HGD groups in the training set. In testing set NNET differentiated NDBE from LGD in 89% of the cases (80% of NDBE and 91.7% of LGD) and to differentiate LGD from HGD in 85.7% of the cases (71.4% of LGD and 100% of HGD).
Tomita N. et al. (2019)^163^	Not mentioned	Biopsy specimens	HE	180 whole‐slide images (116 images – training set, 64 – testing set) separated into 379 images	—	convolutional neural network ResNEt‐18 and a grid‐based attention network ImageNet	Normal NDBE Dysplastic BE EAC	Not mentioned	Classification accuracies of attention‐based model were 0.85 (95% CI, 0.81–0.90) for the NDBE class, 0.89 (95% CI, 0.84–0.92) for dysplastic BE class, and 0.88 (95% CI, 0.84–0.92) for the EAC class. The proposed model achieved a mean accuracy of 0.83 (95% CI, 0.80–0.86) and outperformed the sliding window approach on the same testing set.
Critchley‐Thorne R.J. et al.^165^	366 (41 progressors and 142 nonprogressors ‐ training; 38 progressors and 145 nonprogressors ‐ validation)	Biopsy specimens	HE p16 AMACR p53 CD68 COX‐2 CD45RO HIF1a HER2/neu K20	—	—	TissueCypher Image Analysis Platform (Cernostics, Inc.)	Low, interme‐diate or high risk of progression	Expression and co‐expression of markers	15‐feature classifier was developed to predict progression (AUROC 0.804). HRs were 2.45 (95% CI, 0.99–6.07) for the comparison of the intermediate‐risk versus low‐risk group and 9.42 (95% CI, 4.61–19.24), for high‐risk versus low‐risk. NPV 0.98, PPV 0.26.
Frei N.F. et al.^166^	76 (38 progressors and 38 nonprogressors)	Biopsy specimens	HE p16 AMACR p53 CD68 COX‐2 CD45RO HIF1a HER2/neu K20	—	—	TissueCypher Image Analysis Platform (Cernostics, Inc.)	Low, interme‐diate or high risk of progression	Expression and co‐expression of markers	Evoluation of additional spatial biopsy levels from the baseline endoscopy increased the detection rate of progressors by 63.5% (from 30.4% to 49.8%; P 5 0.016). Evaluation of the highest scoring of all biopsies from the baseline and pre‐baseline endoscopies led to an additional increase of the detection rate by 37.6% (from 49.8% to 68.5%, nonsignificant). Annual rate of progression in NDBE patients of high risk was comparable to progression risk in LGD (6.9%).
Davison J.M. et al^167^	268 (58 progressors and 210 nonprogressors)	Biopsy specimens	HE p16 AMACR p53 CD68 COX‐2 CD45RO HIF1a HER2/neu K20	—	—	TissueCypher Image Analysis Platform (Cernostics, Inc.)	Low, interme‐diate or high risk of progression	Expression and co‐expression of markers	High‐risk group had 4.7‐fold increase in risk for HGD/EAC compared to the low‐risk group (95% CI 2.5–8.8, *p *< 0.0001). Patients with NDBE in high‐risk group progressed at a higher rate (26%) than patients with LGD (21.8%) at 5 years.
Diehl D.L. et al.^168^	60 patients	Biopsy specimens	HE p16 AMACR p53 CD68 COX‐2 CD45RO HIF1a HER2/neu K20	—	—	TissueCypher Image Analysis Platform (Cernostics, Inc.)	Low, interme‐diate or high risk of progression	Expression and co‐expression of markers	TissueCypher results influenced 55.0% of management decisions. In 21.7% of patients, the test upstaged the management approach, and in 33.4% of patients the test downstaged the management. .

^a^ HE, hematoxylin and eosin.

^b^ ImCA, intramucosal adenocarcinoma.

*TissueCypher*. TissueCypher (Cernostics, Inc.) is a tissue system pathology assay using set of immunofluorescent markers (p16, AMACR, p53, CD68, COX‐2, CD45RO, HIF1a, HER2/neu and K20). Quantitative integrated image analysis of expression and co‐expression of these markers in combination with morphological changes in nuclei in biopsy specimens of distal esophagus was used to develop a risk assessment model based on 15 parameters that allows identifying patients with low, intermediate and high risk of neoplastic progression.^165^ TissueCypher result predicts progression independently of pathology analysis, segment length, age, sex or p53 overexpression. Use of TissueCypher in patients with NDBE is of great interest: rate of progression in high‐risk patients established by TissueCypher is comparable to rate of progression in patients with LGD.^166^, ^167^ These results allow us to choose personalized treatment for patients with BE. In a prospective study, TissueCypher result influenced management decisions for choosing surveillance interval or method of treatment (endoscopic eradication therapy) in 55% cases.^168^

## 8 IMPORTANT MOLECULAR AND GENETIC EVENTS ASSOCIATED WITH NEOPLASTIC PROGRESSION IN BE

**FIGURE 15:**
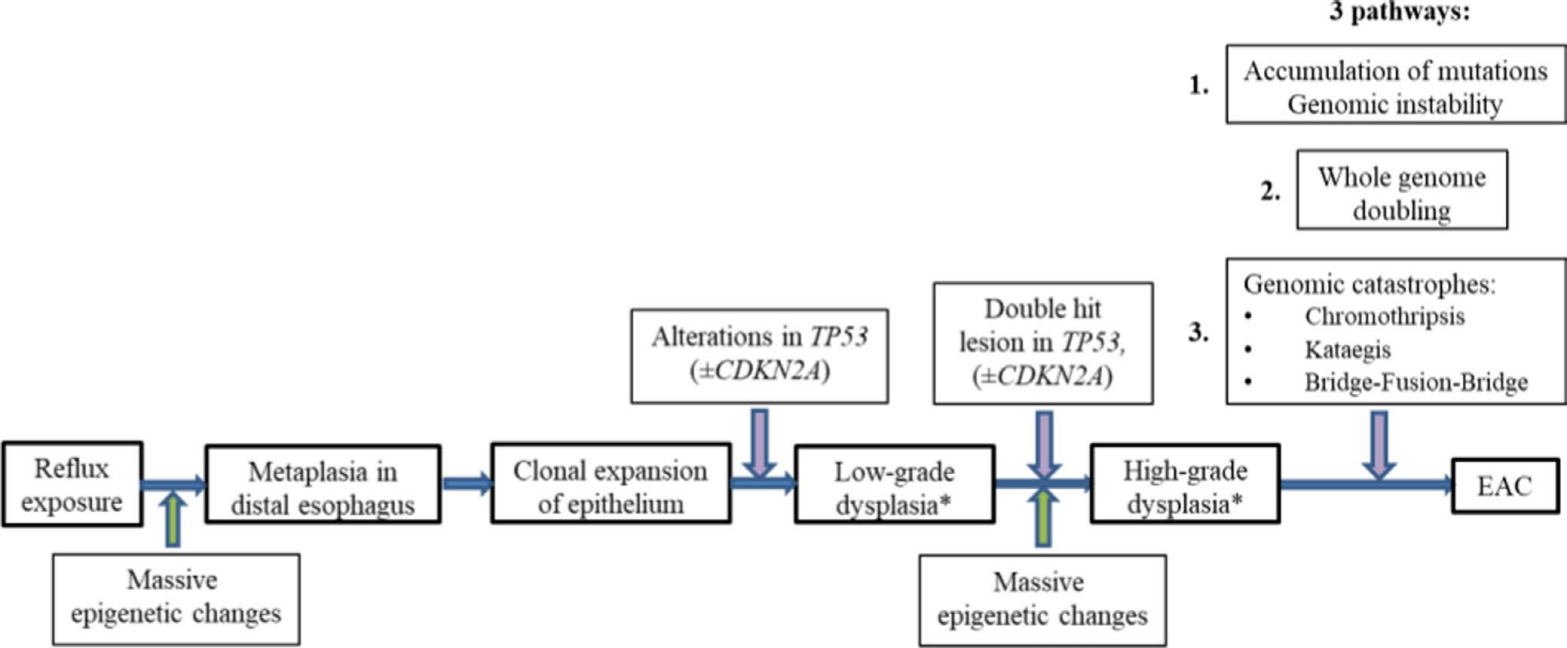
Schematic illustration of the most common genetic events during carcinogenesis in the distal esophagus: * ‐ all aforementioned genetic aberrations were detected in progressors as early as 2 years before EAC diagnosis, place of LGD and HGD at the scheme is elusive

NDBE and especially EAC are marked by high mutational load, surpassed only by lung cancer and melanoma.^169^, ^170^, ^171^ Patients with NDBE who further progress to EAC (progressors) have initially higher mutational load than patients with NDBE who remain stable (non‐progressors).^130^ Various genetic alterations were described in BE and EAC including point mutations, losses of heterozygosity (LOH), as well as large genomic rearrangements, namely, chromothripsis, kataegis and bridge‐fusion‐bridge (BFB) along with aneuploidy and tetraploidy.^3^ Some genetic alterations happen irrespective of carcinogenesis stage, but several genetic events tend to occur at a particular stage of neoplastic progression (Figure 15).

*Loss of heterozygosity in BE and EAC*. LOH is a chromosomal event that leads to deletion of the whole gene and adjacent area at one chromosome requiring transcription from other chromosome containing mutant or inactivated gene. The most common LOHs in BE and EAC include LOH in locus 9p21 (involving gene *CDKN2A*) and locus 17p13 (*TP53*).^129^ Majority of patients with HGD display mosaic of clones and subclones with different patterns of LOH.^172^ Inactivation on *CDKN2A* serves as the earliest, initiating event in pathogenesis of dysplasia and EAC. Although *CDKN2A* inactivation was identified both in patients with dysplasia/EAC and NDBE.^129^ Selective sweep of lesions in *CDKN2A* caused by 9pLOH, promotor methylation or mutation followed by second event in *CDKN2A* or *TP53 (*17pLOH or mutation) is implemented during BE carcinogenesis.^173^ Generating of clones with *TP53* mutations within segment of metaplasia in distal esophagus is key event of progression, leading to increment accumulation of mutations. Mutations in *TP53* are identified in 72%–82.6% of EAC^170^, ^171^; they may arise long before morphological detection of dysplasia in progressors and are seen only in 5% of non‐progressors.^130^, ^169^

*Large genomic rearrangements*. Maley C.C. et al.^174^ demonstrated that patients with more clonal diversity at segment of BE progress more frequently. Generally non‐progressors display small localized deletions in fragile sites and 9pLOH without copy number alterations. In contrary, progressors at 24 months before diagnostics of EAC show huge clonal diversity at segment with large genomic rearrangements including multiple losses and gains as well as whole genome doubling (WGD).^175^ Stachler M.D. et al.^169^ revealed that *TP53* mutations result in rapid WGD followed by genomic instability and oncogene amplification in tumor cells. It is worth mentioning that pathway of WGD is accomplished more often (in 62.5% of EAC) than classical pathway of gradual accumulation of mutations.

Among oncogene amplifications *SMAD4* is remarkable. *SMAD4* gene product forms complexes with other SMAD family proteins and regulates TGFβ‐dependent transcription. New generation sequencing revealed that *SMAD4* mutations are identified only in patients with EAC that may help to distinguish HGD from EAC.^176^

Third pathway of neoplastic progression in BE involves genomic catastrophes.^177^ Whole genome sequencing samples with EAC identified that large genomic rearrangements may result in oncogene amplification through chromothripsis with generation of double‐minute chromosomes (*MYC* и *MDM2*), kataegis or BFB (*KRAS*, *MDM2* и *RFC3*).^178^

*Chromothripsis*. Chromothripsis represents a catastrophic event during carcinogenesis with large‐scale genomic rearrangements including chromosome shattering, gains and losses involving several genes at once and may lead to rapid oncogene activation and inactivation of tumor suppressor genes.^179^ Rausch T. et al.^180^ revealed that chromothripsis is associated with *TP53* gene mutations in children with Sonic‐Hedgehog medulloblastoma caused by Li‐Fraumeni syndrome. Authors proposed three mechanisms contributing to chromothripsis in patients with *TP53* mutations: (1) critical telomere shortening and chromosome end‐to‐end fusion, (2) premature condensation of chromatin due to alteration of cell cycle regulation (i.e., transition from G2 to M phase), and (3) impaired DNA reparation and apoptosis induction. High rate of *TP53* mutations and telomere shortening in EAC elucidate chromothripsis being identified in 30%–32.5% cases.^178^, ^181^ Chromothripsis quiet commonly coincide with kataegis.

*Kataegis* means hypermutation pattern of clustered C > T and C > G at TpC dinucleotides, that was first described in breast cancer.^182^, ^183^ Kataegis arises as a result of APOBEC protein activity that serves as catalytic component of an RNA editing complex. DNA mutator activity of APOBEC is due to C‐to‐U deamination.^184^ In cytoplasm APOBEC restricts replication of DNA‐viruses, including HIV, and comprises a component of natural retroviral defense.^185^ APOBECs predominantly target single‐stranded DNA, and can produce a cluster of strand coordinated mutations that affect cytosine bases in the same strand. Kataegis is detected at the breakpoints of chromothriptic rearrangements caused by telomere crisis.^186^ So the reason for kataegis is breakage of chromatin bridges of dicentric chromosome by 3'repair exonuclease 1 (TREX1) with generation of single strain DNA acting as a substrate for APOBEC deaminases. Kataegis is diagnosed in 31%–86% observations of EAC.^178^, ^181^

*BFB (break*‐*fusion*‐*bridge) cycles* are initiated by telomere loss followed by fusion of unprotected ends of chromosomes or sister chromatids.^187^ These chromosomes then rupture in anaphase. This process may repeat during several cell cycles resulting in inverted duplications with high copy number alterations. Tumor growth activation originates when these amplified areas involve oncogenes. BFB is detected in 27.3% of EAC and leads to amplification of potent oncogenes (RCF3, MDM2, VEGFA, BCAT1 и KRAS) through double‐minute chromosome generation.^178^ These data give evidence that genomic catastrophes are important in neoplastic transformation of BE and represent an alternative mechanism of malignization. Genomic catastrophes that are often seen in HGD and EAC may probably result in rapid progression.^177^, ^178^, ^181^, ^188^

*Aneuploidy*. Aneuploidy is defined as abnormal number of chromosomes in cell. Using flow cytometry, Rabinovitch P.S. et al.^189^ detected aneuploidy in tumor cells of EAC and in epithelial cells adjacent to tumor. Later Rabinovich P.S. et al.^190^ developed cut‐points to assess aneuploidy (>2.7N) and tetraploidy (4N > 6%) in BE in order to predict progression to EAC. In a retrospective study of biopsy archives of patients with EAC for a 9‐year period, it was shown that DNA ploidy anomalies were detected more often in more advanced lesions (NDBE—13%, LGD—60%, HGD—73%, EAC—100%).^191^ Reid B.J. et al.^129^ proposed that aneuploidy is a late event in EAC development that happens after 17pLOH or *TP53* mutation. It was further proved that aneuploidy and/or tetraploidy in clones with 17pLOH is associated with progression to EAC.^192^

In research of Sikkema M. et al.,^137^ univariate analysis showed that aneuploidy, strong Ki67 overexpression and moderate p53 overexpression were all associated with increased risk of progression to HGD/EAC. Although multivariable analysis revealed that in the presence of LGD, p53 overexpression, and to a lesser extent, Ki67 overexpression remained important risk factors for neoplastic progression, whereas aneuploidy was no longer predictive. Nevertheless, detection of aneuploidy in patients with NDBE long time before progression makes it a plausible biomarker for identifying patients at‐risk of progression.^193^ Thus, Killcoyne S. et al.^194^ demonstrated that genomic copy number abnormalities may appear 10 years before dysplasia detection in BE and are strong predictors of neoplastic transformation. Recently, Douville C. et al.^195^ proposed a method of assessment of aneuploidy in esophageal brushings that identifies early and late chromosomal lesions specific for neoplastic progression in BE.

## 9 EPIGENETIC MARKERS OF BE NEOPLASIA AND PREDICTORS OF PROGRESSION

Epigenetic changes begin at early stages of neoplastic transformation and are regarded as potential predictive markers of progression. Several epigenetic changes are implemented during carcinogenesis^196^, ^197^: (1) DNA methylation, (2) posttranslational modifications of histones, (3) specific miRNAs and (4) nucleosome positioning. In our review, we will mostly focus on DNA methylation and miRNA expression, as these processes were extensively studied in BE and EAC.

*Methylation of DNA*. DNA methylation is performed by DNA methyltransferases (DNMTs) at 5‐position of cytosine, usually dinucleotide sequence CpG serves as a substrate for DNMTs. Most of CpG in mammalian cells are methylated except for CpG islands enriched by CpG sequenced, which are located in promotor regions of 60%–70% genes. Aberrant methylation of CpG islands in carcinogenesis usually results in silencing of gene expression, whereas methylation of CpG sequences outside of promotor regions (gene body methylation), in contract, leads to transcriptional activation of corresponding genes.^197^ DNA methylation is the most studied epigenetic feature associated with neoplastic progression in BE. Not only hypermethylation of CpG islands,^198^, ^199^, ^200^ but also hypomethylation outside of them serves as epigenetic hallmark of progression.^200^, ^201^ Thus, Alvarez H. et al.^201^ showed significant genome‐wide hypomethylation in NDBE compared to squamous epithelium; second shift toward hypomethylation was seen in HGD and EAC. Widespread hypomethylation was associated with transcriptional activation of *XCL1*, *XCL3*, *GATA6* and *DMBT1*. In accordance, Xu E. et al.^200^ demonstrated decreased DNA methylation level outside of CpG islands and increased methylation in CpG islands in patients with BE and EAC compared to squamous epithelium. These coexisting epigenetic phenomena cause global changes of transcriptome that are involved in EAC development and appear early in carcinogenesis. Hypermethylation of genes *SFRP1*, *GBX2*, *ADAM12*, *PTGDR*, *DMRT1*, *PTPRT*, *SH3GL3*, *LAMA1*, *COL5A1* and *AJAP1*, that were identified in cancers of other locations, was seen in BE as well as in EAC.^200^ In retrospective study hypermethylation of *CDKN2A*, *RUNX3* and *HPP1* was identified in patients with BE 2 years before EAC diagnostics and was associated with increased risk of progression.^198^ Based on methylation index of these genes, pathomorphological features and segment length authors developed three‐tiered risk stratification model to predict progression in BE.^202^

Alvi et al^199^ studied methylation of imprinted genes and genes located on X chromosome in patients with BE and EAC. They detected 4 genes (*SLC22A18*, *PIGR*, *GJA12* and *RIN2*) differently methylated in NDBE, dysplastic BE and EAC (AUC = 0.988). In a prospective cohort of patients, methylation of less than 2 genes was seen in patients with low risk of progression to EAC, and methylation of 2 genes was associated with intermediate risk and >2 genes – with high risk of EAC development.

Kaz A.M. et al.^203^ identified 4 unique methylation profiles in BE and EAC: BE with low and high methylation epiphenotype and EAC with low and high methylation epiphenotype. Authors also found 17 differently methylated sites of CpG (differently methylated positions [DMPs]) that may distinguish BE and EAC and 3 DMPs for NDBE and HGD. Yu M. et al.^204^ showed that high methylation is associated with mutations or amplification of *ERBB2*, and also harbors higher mutational load. Moreover, authors revealed that cell lines with different DNA methylation level are characterized by different sensitivity to drugs (SN‐38, topotecan and palbociclib). Therefore, assessment of DNA methylation level is useful for indication of target treatment. Jammula S. et al.^205^ also defined 4 subtypes of patients with BE and EAC based on DNA methylation intensity. Patients with 1 subtype showed DNA hypermethylation with high mutational load and mutations in cell cycle controlling genes (*CCND1*, *CCNE1*, *MYC*, *CDK6*) and receptor tyrosine signaling pathways (*GATA4*, *ERBB2*, *KRAS*). Subtype 2 consisted predominantly of patients with BE with upregulation of transcriptional factors HNF4A/G, FOXA1/2/3, GATA6 and CDX2, as well as high expression of genes associated with ATP synthesis and fatty acid oxidation. Patients of subtype 3 did not show changes in methylation pattern, compared with control tissue, but displayed heavy inflammatory infiltration enriched with cytotoxic cells, B‐cells, mast cells and neutrophils along with cancer associated fibroblasts and reduced levels of T‐helper cells. Subtype 3 was associated with the lowest survival, whereas the highest survival was expectedly found in subtype 2. At last, patients with subtype 4 showed hypomethylation accompanied with large‐scale genomic rearrangements, copy number alterations and amplification of *CCNE1* and *ERBB2*.

Number of DMPs varied in squamous epithelium and BE as well as in BE and EAC is tremendous. Li D. et al.^206^ identified 12 from 458 DMPs that are valuable in distinguishing of squamous epithelium, BE, EAC and esophageal squamous carcinoma and found 3 CpG sites in EAC and 2 CpG sites in esophageal squamous cell carcinoma (ESSC), methylation of which was prognostic (associated with survival). After detection of 257 DMPs, specific for EAC, Peng W. et al.^207^ developed a model for early diagnostics of EAC based on 4 DMPs (cg07589773, cg10474350, cg13011388 and cg15208375, localized in *IKZF1*, *HOXA7*, *EFS* and *TSHZ3*, AUC = 0.903).

In all aforementioned studies, DNA was derived from biopsy samples of distal esophagus, although several non‐invasive methods were proposed for detection of *TFPI2*,^208^ *VIM*,^209^ *CCNA1* и *VIM* ^210^ methylation for BE diagnostics.

*Posttranslational modifications of histones*. Histone modifications regulate gene transcription as well as replication and DNA repair.^196^ Among posttranscriptional modifications, imbalance between acetylation and deacetylation of histones was shown to be implicated in cancer development^211^ and particularly in esophageal carcinogenesis.^212^ Acetylation of lysine residues' by histone acetyltransferases (HATs) results in the relaxation of DNA structures and facilitates gene transcription, whereas hypoacetylation of histones is a hallmark of inactive heterochromatin. Cancer cells are characterized with impaired balance between HATs and histone deacetylases (HDACs) which severely alters chromatin structure and, as a consequence, alter gene expression, including genes, involved in the cell cycle regulation, differentiation and apoptosis.^211^ For example, HDACs repression causes hyperacetylation of histones which increases transcriptional activity, including rise in expression of potent oncogenes, initiating carcinogenesis.^211^ On the other hand, HDACs overexpression leads to histone hypoacetylation and impaired cell cycle (increase in cyclin dependent kinases 2 and 4 and abundant phosphorylation of retinoblastoma protein) that results in augmented cellular proliferation.^210^ HDACs inhibitors are valuable novel anti‐cancer drugs that arrest tumor growth, promote apoptosis,^211^ help us to overcome chemotherapy resistance and increase reactive oxygen species, causing DNA and membrane damage in cancer cells.^212^ Moreover, HDACs inhibitors impair miRNA expression showing huge interaction between different epigenetic modifications.^213^

Like posttranslational modifications of histones, *nucleosome positioning* modulates accessibility of regulatory DNA sequences for transcriptional factors.^196^, ^214^ Specific information about nucleosome positioning and its close interaction with DNA methylation is provided in several papers.^214^, ^215^, ^216^, ^217^

*miRNA*. miRNAs are small noncoding sequences of 20–25 nucleotides that maintain posttranscriptional regulation of target genes. MiRNAs express tissue‐specific way and control wide spectrum of biological processes, including proliferation, apoptosis and differentiation.^218^ Numerous data comparing miRNA expression profiles in tumors and corresponding normal tissues demonstrate widespread changes in miRNAs expression during carcinogenesis.^218^, ^219^ MiRNAs function either as tumor suppressors or as oncogenes, depending on target genes.

Maru D.M. et al.^220^ showed that increased level of miRNA‐196a in biopsy samples of distal esophagus is a potential biomarker of progression from NDBE to EAC, therein expression of target genes (*SPRR2C*, *S100A9* and *KRT5)* falls rapidly through neoplastic transformation. Fassan M. et al.^221^ revealed different miRNA expression profiles of esophageal squamous epithelium, IM without dysplasia, LGD, HGD and EAC. Authors detected increase in miR‐215 and miR‐192 accompanied by decrease in miR‐205, miR‐203 and let‐7c levels during carcinogenesis. In prospective research Revilla‐Nuin B. et al.^222^ identified, that elevated levels of 4 miRNAs (miR‐192, 194, 196a and 196b) are associated with progression to EAC. Many other miRNAs involved in neoplastic progression in BE were identified.^223^, ^224^, ^225^ In meta‐analysis miR‐192, miR‐194, miR‐203, miR‐205 and miR‐215 were found to be perspective tissue biomarkers for BE diagnosis.^226^

MiRNAs are also used in non‐invasive diagnostics of BE, e.g., using Cytosponge (combination of miR192, miR196a, miR199a and TFF3).^227^ Circulating miRNAs of plasma may also serve as a diagnostic sample.^228^, ^229^, ^230^, ^231^ For example, Bus P. et al.^228^ validated combination of circulating miRNA for differential diagnostics of BE and EAC. In addition, level of miR130a increased gradually in line NDBE—LGD—HGD—EAC stage I, II—AКП stage III, IV.^231^

Value of miRNAs in diagnostics is obvious (Table 7), besides levels of specific miRNAs may serve as prognostic markers and are also applicable for assessment of treatment efficacy and as therapeutic targets.^232^, ^233^, ^234^

**TABLE 7:** Overview of miRNA, associated with neoplastic progression in BE

Advantages	Disadvantages	Markers, elevated with progression	Markers, decreased with progression
Personized diagnostics Capability to use different specimens (biopsy pieces, Cytosponge brushing,^227^ plasma,^228^ serum^225^, ^229^, ^230^, ^231^) Potential tool for prognosis and assessment of treatment efficacy.^232^, ^233^, ^234^ May represent a therapeutic target.	Ongoing search for clinically relevant and cost‐effective markers of progression. Need for validation of novel markers in clinical trials.	↑miR‐21^223^, ^224^ ↑miR‐25^223^, ^224^ ↑miR‐92a‐3p^230^ ↑miR130a^231^ ↑miR‐136‐5p^228^ ↑miR‐192^221^, ^222^, ^227^ ↑miR‐194^222^, ^232^ ↑miR196a^220^, ^222^, ^224^, ^227^ ↑miR‐196b^222^ ↑miR‐199a^227^ ↑miR215^221^ ↑miR‐223^223^ ↑miR‐301b^223^ ↑miR‐382‐5p^228^ ↑miR‐618^223^ ↑miR‐17‐92 cluster^223^	↓let‐7c^221^, ^223^ ↓miR‐23b^223^ ↓miRNA‐133a‐3p^228^ ↓miR‐199a‐3p^229^ ↓miR‐203^221^, ^223^, ^224^ ↓miR‐205^221^, ^223^, ^224^ ↓ miR‐320e^229^ ↓miR‐375^223^ ↓miR‐378^224^

Epigenetic changes are the earliest in pathogenesis of BE, anticipating any genetic or molecular alterations during Barrett's carcinogenesis. Several epigenetic changes serve as stage‐specific markers of neoplastic transformation which is important for precise diagnosis. DNA methylation and miRNA profiles are promising tools for non‐invasive diagnostics of BE and EAC. Moreover, epigenetic alterations provide new targets for treatment.

## 10 MICROENVIRONMENT MARKERS IN PROGRESSION TO BARRETT'S ADENOCARCINOMA

Microenvironment during carcinogenesis can be divided into 3 dynamic stages: tumor precursor microenvironment, tumor microenvironment (TME) and pre‐metastatic niche.^235^ TME consists of adaptive and innate immune cells, fibroblasts, adipocytes, endothelial cells and extracellular matrix (ECM) components.

Chronic inflammation, caused by gastric and bile acid reflux, results in recruiting of immune cells and releasing a variety of mediators (e.g. IL‐1β, IL‐8 and IL‐6), which together establish BE microenvironment that favors dysplasia initiation and further development of EAC.^235^, ^236^, ^237^, ^238^ Numerous immune changes in BE were associated with progression to EAC. Flow based single cell analysis showed that B cell rich microenvironment in normal esophagus changes into predominantly T cell rich landscape in BE.^239^ Using IHC evaluation, Porter et al.^240^ revealed that NDBE is associated not only with reduced lymphocytic infiltration of CD20+ B‐cells, but also with lower level of CD4+ T‐cell and CD8+ T‐cell infiltration compared with squamous epithelium of esophagus. In this study dysplastic BE demonstrated an increase of CD20+ B‐cells, CD8+ T‐cells and Foxp3+ Tregs compared with NDBE. Importantly, individuals with dysplasia also showed increased CD20 + B‐cells in background NDBE compared with nonprogressors, and patients with EAC displayed increased CD20+, CD4+ and CD8+ lymphocytes in the background NDBE compared with nonprogressors. In rat model Miyashita T. et al.^241^ showed that M2 phenotype CD163+ macrophages (tumor‐associated macrophages, TAMs) infiltration contributes to tumor development along with Foxp3+ Tregs via Stat3‐pathway.

Kavanagh ME et al.^242^ demonstrated Th2 phenotype in BE, characterized by elevated levels of IL‐4 producing CD4+ T‐cells and secreted levels of IL‐6, and immunocompromised T‐cells infiltrating EAC with low expression of CD45RO and CD69 that facilitate tumor progression and may represent a target for immune therapy. The same researchers identified that circulating T cells in EAC patients exhibited impaired migratory capacity with decreased frequencies of Th1‐associated CXCR3+ and Th17‐associated CCR6+ cells.^243^ Interestingly, neutrophil‐lymphocyte ratio (NLR) in blood gradually increased from NDBE to EAC. NLR >2.27 was able to diagnose EAC with 80% sensitivity and 71% specificity (area under the curve = 0.8).^244^

RNA‐Seq and the genomic cellular analysis tool xCell revealed a linear increase in Th1, Th2, Treg, and pro–B cell populations in EAC compared with precancerous lesions (dysplastic BE and NDBE) as well as a linear increase in M1 and M2 macrophages between HGD and EAC.^245^ Although multiplex IHC showed that immune cell populations tended to increase in a stepwise fashion from BE to LGD to HGD, followed by a decline in all evaluated immune cell populations in EAC tissues that coincided with increased PD‐L1 expression.^245^ PD‐L1 has been shown to cause T cell apoptosis and suppress antitumor immunity.^246^, ^247^ PD‐L1 expression in subset of EAC patients means that these individuals may benefit from immunomodulatory therapy, such as anti–PD‐1, anti–PD‐L1 or anti‐CTLA4 therapy.

Changes of the ECM in the BE microenvironment also are important in carcinogenesis. Matrix metalloproteinases (MMPs) are components of ECM involved in inflammation and tumor metastasis. IHC showed that MMP‐7 was weakly expressed in squamous epithelium adjacent to EAC but increased progressively in epithelial cells in NDBE, LGD, HGD and EAC, particularly at the invasive front.^248^ Moreover, MMP‐7 was weakly expressed in the stroma myofibroblasts of dysplastic BE and EAC, especially at the invasive front. Authors supposed that MMP‐7 in BE epithelial cells was regulated by PI3‐K kinases and could stimulate stromal cell migration, invasion and remodeling of the microenvironment.^248^ MMP9 and MMP13 are also up‐regulated in BE.^249^ Expression of MMP13 was higher in NDBE, whereas expression of MMP‐9 was higher in EAC. Herszenyi L et al.^250^ demonstrated that MMP9 expression level gradually increased from NDBE to EAC making MMP9 a prognostic biomarker. Wang Z et al.^251^ demonstrated that expression levels of *COL1A2* (encoding α2 chain of collagen I) and related genes (*COL1A1*, *COL3A1*, *ZNF469*, and *POSTN*) were positively correlated with the infiltration levels of macrophages and dendritic cells, and the expression levels of *ZNF469* was also positively correlated with the infiltration levels of CD4+ T cells in both EAC and ESCC. These results indicated these genes might be the candidate genes for assessing the immune infiltration levels in esophageal cancer. COL1A2 is known to play a role in the invasion and metastasis of ovarian cancer.^252^ COL1A2 also up‐regulates proliferation, migration and invasion of ESCC *in vitro*.^253^

Changes in different immune cell populations as well as components of ECM are elucidated across the progression from BE to EAC. Some of immune changes are of value because they represent targets for immunomodulatory treatment. A lot of novel markers associated with BE progression to EAC are identified in scientific studies and need to be evaluated in clinical trials before becoming part of the routing diagnostics.

## 11 CONCLUSIONS AND OUTLOOK

Endoscopic examination with morphologically confirmed IM is a standard of BE diagnostics. Morphological verification of dysplasia is challenging and provides great variability in diagnosis. In difficult cases IHC evaluation is reasonable. IHC examination with p53, Ki67 and AMACR not only allows identifying presence and grade of dysplasia, but also has implication in determining prognosis. TissueCypher technology provides quantitative analysis of epithelial and stromal immunofluorescent markers expression (p16, AMACR, p53, CD68, COX‐2, CD45RO, HIF1a, HER2/neu and K20) in biopsy specimens with BE. TissueCypher results are interpreted in terms of low, intermediate or high risk of progression to EAC.

Population of patients with BE is heterogeneous: although some patients are stable with NDBE, others may rapidly evolve to dysplasia and EAC. Analysis of genetic and epigenetic alterations in BE and EAC sheds light on pathways of neoplastic progression in distal esophagus and gives a key to stratification of progression risk in each individual patient, meaning that molecular and genetic alterations arise earlier than morphologically identifiable dysplasia. Noninvasive detection of epigenetic markers of BE and EAC or detection of markers in plasma or serum of patients is a promising alternative to EGS with biopsy and is valuable for diagnosis, progression and survival prognosis and assessment of therapy efficacy.

## CONFLICT OF INTERESTS

The authors declare no conflicts of interest in the writing and preparation of this article.

## AUTHOR CONTRIBUTIONS

K.M., A.D., D.A., M.S., L.M. developed the methodology and developed the goals and main criterion for the project. K.M., A.K. and M.S. gathered the methodology, gathered literature, and prepared primary drafts. M.S., K.M., and L.M. analyzed the primary results and wrote the primary article. K.M., A.D., D.A. and M.S. prepared the manuscript. All authors reviewed the final manuscript.

## Data Availability

All associated data are available from the corresponding author upon reasonable request.
